# Effect of extrusion on available energy and amino acid digestibility of barley, wheat, sorghum, and broken rice in growing pigs

**DOI:** 10.5713/ab.23.0285

**Published:** 2023-11-01

**Authors:** Ge Zhang, Gang Zhang, Jinbiao Zhao, Ling Liu, Zeyu Zhang

**Affiliations:** 1 State Key laboratory of Animal Nutrition, College of Animal Science and Technology, China Agricultural University, Beijing 100193, China

**Keywords:** Cereals, Digestible Energy, Extrusion, Growing Pigs, Metabolizable Energy

## Abstract

**Objective:**

The main objective of this study was to determine available energy and nutritional digestibility of extruded cereals and the effect of extrusion on the nutritional value of feed ingredients, aiming to provide scientific basis for efficient application of extrusion in the diets of growing pigs.

**Methods:**

In Exp. 1, 48 crossbred growing pigs (Duroc×Landrace×Yorkshire) with an initial body weight (BW) of 34.6±2.2 kg were selected and fed with eight diets (non-extrusion or extrusion) to determine the digestible energy (DE), metabolizable energy (ME), and nutrients digestibility. Eight diets included extruded grains (barley, wheat, sorghum, or broken rice), while four had unprocessed grains. In Exp. 2, 9 diets were formulated including 4 cereals with extrusion or non-extrusion and a N-free diet. In addition, 9 growing pigs (BW = 22.3±2.8 kg) were fitted with T-cannula in the distal ileum and arranged in a 9×6 Youden square design.

**Results:**

Results show that apparent total tract digestibility of gross energy, dry matter, organic meal, ether extract, neutral and acid detergent fiber was not affected by the extrusion process and there was no interaction between cereal type and extrusion treatment on DE, ME. However, the apparent total tract digestibility for crude protein (CP) increased markedly (p<0.05). The standardized ileal digestibility (SID) of all amino acids (AA) except for leucine remarkably increased by extrusion (p<0.05). There was an interaction on the SID of arginine, leucine, isoleucine, methionine, phenylalanine, cystine, and tyrosine in growing pigs between type of grain and extrusion treatment (p<0.05).

**Conclusion:**

Extrusion increased the ileal digestibility of CP and most AA in cereals, however, the DE and ME of cereals were not affected in growing pigs.

## INTRODUCTION

Starch from grains including corn, rice, wheat, barley, sorghum is the primary source of energy in pig diets [1]. Differences in the carbohydrate composition of cereals often affect the nutrient digestibility in the diet [2], gut health and growth of pigs [3]. According to previous studies, endogenous enzyme activity in piglets increased progressively with age. Low alpha-amylase activity might limit absorption and utilization of natural starch from diets fed to piglets [4]. Extrusion can increase the degree of starch gelatinization of cereals and improve nutrient digestibility by increasing the contact area between enzymes and cereals [5,6]. White et al [7] have proved that extrusion improves the digestibility of starch *in vitro* and the digestibility of starch in piglets, which mutually confirm the beneficial effect of extrusion on piglets. Therefore, extrusion would be a good technique to improve the quality of piglets’ diets. However, the funding of this effect in growing pigs remains to be discovered.

Extrusion may enhance the apparent total tract digestibility (ATTD) of gross energy (GE) and starch, mainly relying on the nature of the cereals and that value may present different depending on the degree of starch or dietary fiber [8]. Previous researchers reported the average daily gain (ADG) and feed conversion rate (FCR) of weaned piglets fed an extruded grains diet increased by and decreased by 8% and 6%, respectively [6]. Extrusion of sorghum improved GE and nitrogen digestibility, but did not ADG and FCR [9]. Herkelman et al [10] reported extruded corn did not affect the utilization of lysine in finishing pigs, but provided higher digestible energy (DE) and metabolizable energy (ME) than non-extruded corn. The ATTD of crude protein (CP) was increased by the extrusion process, and digestibility of ether extract (EE) tended to show a decrease with extrusion [11]. However, other studies have shown that extruding wheat significantly decreased the FCR of piglets but did not change in ADG and average daily feed intake of piglets [12,13]. The data published so far show that the type of grain or the characteristics of the grains have the greatest influence on the product, and secondly, the extrusion conditions. It has been shown that high temperatures lead to the Maillard reaction, which is thought to cause a decrease in proteins and amino acids (AA) digestibility [14, 15].

The objective of this study was to evaluate the effect of extrusion on nutritional value of cereals. Barley, wheat, sorghum, and broken rice were selected to explore the nutrient composition, DE, ME, and nutrient digestibility of cereals with and without extrusion.

## MATERIALS AND METHODS

The study was conducted according to the guidelines of the Declaration of Helsinki and approved by the Institutional Animal Care and Use Committee of China Agricultural University (CAU AW01102202-1-1, Beijing, China). The experimental regulations and methods were approved and then performed according to relevant criteria.

### Raw materials and extrusion treatment

Four types of cereals (barley, wheat, sorghum, and broken rice) were purchased (Shennong feed Technology Co., Ltd. Henan, China). The extrusion process was as follows: the equipment was a high-capacity twin-screw extruder with a 200 mm diameter barrel and a length/diameter (L/D) ratio of 20 (Yang Gong Extruder, Model TPE62S, Beijing, China). The extruder barrel temperature and the screw speed were controlled by a computer, using parameters recommended by the manufacturer for the swine diet. Specifically, the conditioning temperature was 70°C to 75°C, feed section temperature was 85°C to 90°C, compression section temperature was 125°C to 130°C and extrude section temperature was 130°C to 135°C.

### Animals, dietary treatments and experimental design

In Exp. 1, a total of 48 growing barrows (n = 48) with an initial weight of 34.6±2.2 kg were assigned to 8 dietary treatments (barley, wheat, sorghum and broken rice, extrusion, or non-extrusion) in a randomized complete block design, with 6 pigs per treatment. The experiment lasted 12 days with the first 7 days as an acclimation period and the last 5 days as a sample collection period. The diets were formulated and supplemented with vitamins and minerals to meet or exceed the nutritional requirements of growing pigs (Tables 1 and 2) [16].

In Exp. 2, nine barrows with an initial BW of 22.9±2.2 kg were fitted with a T-cannula at the distal ileum. In a 9×6 Youden square design, pigs were allotted to 9 dietary treatments in 6 collection periods. Animal care and surgical procedures followed the previous study [17] and the T-cannula used here was previously described elsewhere [18]. The experimental diets included an N-free diet and 8 test diets containing extruded or non-extruded cereals (Table 3). Each experiment period lasted 7 days, with the first 5 days being the acclimation period and the last 2 days being the digestive collection period.

### Feeding and sample collection

In Exp. 1, all pigs were housed individually in stainless steel metabolism cages (1.4 m×0.7 m×0.6 m) at a temperature of 18°C to 22°C. According to the recommended dietary requirements of swine, pigs had free access to water and received a daily ration equivalent to 4% of their body weight at 0830 and 1530 [19]. Cages were cleaned and disinfected twice a day to maintain sanitation.

Feces were collected in plastic bags and stored at −20°C. To prevent nitrogen loss in urine, 25 mL of 6 mol/L HCl was added to the plastic collection bucket. After filtering urine with gauze every day, 1% was collected to a plastic bottle and stored immediately at −20°C. Spilled feed and remaining feed were collected, dried, and weighed every day. At the end of the experiment, the collected fecal samples were thawed naturally at room temperature, weighed, and recorded. Samples were dried at 65°C for 72 hours and weighed after 24 hours. Additionally, urine samples collected from each pig were thawed and mixed, filtered again through gauze and approximately 40 mL were removed for analysis. Urine and fecal samples from each pig were stored at −20°C before analysis.

In Exp. 2, after two weeks recovery period, the pig was weighed and housed individually in stainless steel metabolism cages described in Exp. 1. Digesta was collected on days 6 and 7 of each period of the experiment in accordance with the previous report [18]. Smaller collection bags (200 mL) were used for ileal digesta collection, the bag is a sterile bag without adding any substances. According to Huang et al [20] description, timely replacement of new bags and transfer of chyme to −20°C to prevent AA degradation. Samples of ileal digesta were collected at 0800 and 1700 of each day and stored at −20°C.

### Chemical analysis

In Exp. 1, the urine and fecal samples were thawed and then ground non-extruded or extruded cereals, diets, and fecal samples, until they were fine enough to pass through a 1 mm (40 mesh) screen. The GE in samples using bomb calorimetry (Model 6400; Parr Instruments, Moline, IL, USA), dry matter (DM) content according to AOAC method 930.15, CP content following AOAC method 984.13, EE content according to AOAC method 920.39, ash content according to AOAC method 942.05. According to the previously described method [21], filter bags (Model F57; Ankom Technology, Macedonia, NY, USA) and fiber analyzer (ANKOM200 Fiber Analyzer; Ankom Technology, USA) were used to analyze neutral (NDF) and acid detergent fiber (ADF). The insoluble dietary fiber (IDF) and soluble dietary fiber (SDF) contents of cereals were measured by Dietary Fiber Analyzer (Ankom TDF Dietary Fiber Analyzer; Ankom Technology, USA) following [22].

In Exp. 2, ileal digesta was thawed and mixed for each pig, then freeze dried in a vacuum freeze dryer. Dried samples were ground to pass through a 1 mm (40 mesh) screen. The AA content in raw materials, feed, and digesta by AOAC method 982.30 [22].

### Calculation

The OM, total dietary fiber (TDF), DE, ME, and the ATTD of GE, DM, OM, CP, NDF, and ADF in diets were calculated using the previous method [19]. In brief, the direct method was used to calculate the DE and ME of diets. The DE of diets equals total GE intake minus GE content of feces, ME of diets equals DE of diets minus GE content of urine. The DE and ME values of cereals were calculated as the DE and ME values in the corresponding diets divided by 0.969 (the proportion of cereals in the diet). The CP and AA digestibility of cereals was calculated as described by Stein et al [23], because cereals are the only source of CP and AA in the diet, the digestibility of CP and AA in the diet is the same in the cereals.

### Statistical analysis

Data from Experiment were checked for outliers, normality, and homogeneity of variance. using the UNIVERIATE procedure in SAS 9.2 [24]. The data were analyzed as a 4×2 factorial treatment arrangement using the GLIMMIX procedure: grain types, extrusion processing and their interaction were fixed main effects, and experimental period as a random effect. Each pig was analyzed as an experimental unit. Least squares means were calculated using the LSMEANS statement, and a value of p<0.05 was considered as statistically significant, and 0.05≤p<0.10 was considered a trend.

## RESULTS

### Analysis of nutritional component

The DM of wheat and sorghum increased by 5.65% and 5.24%, and the CP of barley and sorghum, increased by 23.49% and 36.00% respectively after extrusion (Table 1). Except for the diets with broken rice, the levels of NDF, ADF, TDF, SDF, and IDF decreased slightly after extrusion, and EE in all cereal samples decreased by about 30%. No SDF was detected in both non-extruded and extruded broken rice.

### Nutrients digestibility and energy content

There were no interactive effects in DE and ME between cereal types and extrusion (Table 4). The DE and ME in broken rice were higher than in other cereals (p<0.05). No differences in interactions on the ATTD of GE, DM, OM, CP, EE, NDF, and ADF in growing pigs between cereal types and extrusion were observed. Extrusion did not affect the ATTD of GE, DM, OM, EE, NDF, and ADF in broken rice, wheat, barley, and sorghum, but the ATTD of CP in the extrusion diets was significantly higher than unextruded treatments (p<0.01). Broken rice (non-extrusion or extrusion) had the highest ATTD of GE, DM, OM, EE, and NDF than other cereals (p<0.01) but was not altered by extrusion.

### Standardized ileal digestibility of crude protein and amino acids

In Tables 5 to 8, there was no interaction between cereal types and extrusion treatment on apparent ileal digestibility (AID) and standardized ileal digestibility (SID) of CP, but the effect of extrusion on AID and SID of Arg, Leu, Ile, Met, Phe, Cys, Lys, Trp, Tyr, Cys, Glu, and Tyr was different among different cereal types (p<0.05). The extrusion treatment increased the SID of CP and most AA except for Leu, and the AID and SID of most AA in broken rice were higher than in other cereal grains (p<0.05). In the experiment, SID of CP, Lys, Met, Thr, Trp, and Val were founded to increase by 7.53, 11.24, 5.24, 9.06, 5.75, and 7.63 percentage units, respectively.

## DISCUSSION

### Effect of extrusion on the available energy and digestibility of cereals

The DE and ME of barley, wheat, sorghum, and broken rice determined in this study were higher than previously reported values [16,25] and this variation may be due to different sources or different extrusion processes. Notably, the EE of the grains decreased after the extrusion processing, and the loss of EE was attributed to the extrusion process that promotes the release of fat into free fatty acids, which combined with starch and protein to create complexes [26]. In experiments, the DE and ME of barley reduced slightly after extrusion, and a higher temperature (174°C) was more suitable for barley extrusion process, because Rodrigues et al [8] reported that extrusion process enhanced the digestion of energy and CP in the weaned piglets' small intestines. Higher temperatures produced more damage to cell walls of barley and increased the utilization of nutrients by digestive enzymes. Unlike barley, DE and ME of broken rice, wheat, and sorghum increased slightly after extrusion process, indicating that the extrusion parameters of this study were more suitable for the latter. In addition, the ATTD of DM and OM were lower in barley compared to wheat, broken rice, and sorghum mainly due to the higher SDF in barley that increased the viscosity of the digesta, which resulting in increased residence time of chyme [27]. A previous report showed that the CP digestibility increased after extrusion of rice, barley, wheat, corn, oats, and sorghum but decreased in rice [8]. Broken rice improved the growth performance of weaned piglets because of its higher ATTD of GE, DM and nitrogen [28], which was also confirmed by our experimental results that showed the highest digestibility of various nutrients in barley, wheat, and sorghum before and after extrusion. One unanticipated result was that ATTD of EE decreased in all four grains, combined with changes in EE, we believe that low dietary EE which leads to high variation. Based on the results mentioned above, the advantages of extrusion may be more pronounced in cereals with low digestible nutrients, such as sorghum.

The result of previous studies on the effect of extrusion on fiber levels in cereal grains were reported as scarce and inconsistent. Zhang et al [29] reported a higher content of SDF in oat bran (14.2%) appeared after extrusion, and in our study, the levels of NDF and ADF in barley decreased slightly after extrusion, but there is a tendency for the improved ATTD of NDF. Jaworski et al [30] reported that the ATTD of DM, CP, GE, ADF, and nitrogen-free extract decreased linearly when the dietary bran level increased from 0% to 30%. In the current study, the extrusion reduced the dietary fiber of the grain and improved the apparent digestibility of grain nutrients. Extrusion reduced contents of NDF, ADF, and IDF in cereal grains, but the effect of dietary fiber on nutrient digestibility needs to be further clarified.

### Effect of extrusion on standardized ileal digestibility of crude protein and amino acids

Results for the effects of extrusion on SID of CP and AA varied due to the discrepancy of cereals and extrusion parameters. Under the heating conditions, the free amino and carbonyls in AA undergo a Maillard reaction, resulting in changes in the AA [31]. In the present study, the extrusion process increased the SID of most AA and CP, so the reduction in NDF and ADF levels in wheat, barley, and sorghum may have contributed to the increased AA digestibility in the cereal grains. An increase in EE in the diets could delay gastric emptying [32], thereby improving AA digestibility in the nursery and growing pigs. The decreased EE value in the ingredients during the present study was inconsistent with previous report [25], we speculate that losses during extrusion may be the main reason. A more accurate method of measuring may be required or acid-hydrolyzed ether extract used as an indicator. Corn and wheat have different proteins that react differently to extrusion [26], the increased content of resistant starch (RS) in extruded grain may affect the synthesis of bacterial proteins in the large intestine and thus alter the apparent digestibility of CP [33,34]. It is noteworthy that the increase in SID of AA by extrusion was greater in barley compared to other cereals. We think that perhaps those grains with lower nutritional value are more suitable for extrusion, just like the presence of tannins in sorghum seriously affects the digestion and absorption of other nutrients [35]. In conclusion, the effects of extrusion on the nutritional value of grains are diverse and the influence of extrusion parameters on the nutritive value of cereal grains needs further study.

### Effect of extrusion on different cereals

In the study, cereal types and extrusion processes had an interactive trend between in DE and ME, the reason for this may be the different concentration of RS and TDF in cereals [36]. According to previous research [16], the starch value (DM) of barley, wheat, sorghum and broken rice were about 56%, 69%, 78%, and 85% respectively, and the TDF value (DM) were about 21.01%, 16.06%, 13.76%, 0.56% respectively. The interactive trend observed for the ATTD of DE and ME may be the result of varying starch and TDF content of the grains. The addition of sugarcane bagasse significantly increased the starch saccharification rate of cassava starch, which may lead to an increase in the number of free hydroxyl groups and swelling capacity in cereals due to the coarse nature of sugarcane bagasse and increased shear force [37]. The different TDF content of grains may be the reason why no interaction was observed. Zhuo et al [11] reported rice with lower non-starch polysaccharide levels and lower amylose-to-amylopectin ratios may contribute to an increased starch digestibility, and similar results were observed in the extruded broken rice group. The effect of extrusion on AID and SID of most AA and CP was different among cereal types and extrusion treatment, which may be associated with that extrusion breaks the connection between CP and fiber in the grain, releasing some CP and AA. The ileal digestibility of DM was improved after corn was extruded, but the difference in AA digestibility was not significant [38]. One reason for the result above may be related to the different parameter settings [25,26], extrude temperature, screw speed, and feeding rate are all important indicators that affect nutrition of cereals. In addition, the extrusion conditions used in previous studies were different from our findings.

## CONCLUSION

Based on the test condition, there is no differences in the interactive effects on the DE and ME for growing pigs between extrusion and cereal types. Extrusion did not affect the DE and ME of cereal grains but increased the digestibility of CP. There were significant differences in DE and ME for different cereal grains. For most AA, there was an interaction between extrusion treatment and grain type, with extrusion treatment significantly increasing the SID of AA. Furthermore, many results under other condition remain unclear and need further research.

## Figures and Tables

**Table 1:** Chemical composition of the experimental ingredients (%, as-feed basis)^1)^

Item	Broken rice		Wheat		Barley		Sorghum	
			
	−	+	−	+	−	+	−	+
GE (MJ/kg)	15.61	15.87	16.41	16.39	16.22	16.51	16.33	16.77
DM	89.17	88.96	89.38	94.43	89.46	91.69	87.60	92.19
OM	88.91	88.58	87.42	92.15	87.25	89.30	86.16	88.43
CP	8.21	8.73	14.65	14.80	7.96	9.83	9.11	12.39
EE	0.54	0.35	1.41	0.97	1.24	0.83	2.58	1.75
NDF	1.53	2.96	18.78	17.01	22.07	19.12	12.62	11.35
ADF	-	-	3.09	2.66	5.88	4.51	7.89	6.98
TDF	0.50	0.20	14.35	13.70	18.80	17.95	12.05	9.80
SDF	-	-	2.30	1.80	3.20	2.95	1.80	1.30
IDF	0.50	0.20	12.05	11.90	15.60	15.00	10.30	8.50
Ash	0.26	0.38	1.96	2.27	2.22	2.39	2.88	3.76
Indispensable AA								
Arg	0.63	0.64	0.59	0.59	0.43	0.43	0.34	0.61
His	0.19	0.20	0.28	0.29	0.19	0.19	0.21	0.30
Leu	0.65	0.67	0.82	0.83	0.57	0.59	1.15	1.36
Ile	0.34	0.35	0.45	0.45	0.30	0.31	0.36	0.54
Lys	0.29	0.30	0.36	0.35	0.35	0.34	0.22	0.48
Met	0.22	0.23	0.21	0.20	0.15	0.15	0.15	0.20
Phe	0.42	0.44	0.55	0.56	0.40	0.41	0.45	0.65
Thr	0.29	0.31	0.36	0.37	0.30	0.30	0.30	0.44
Trp	0.12	0.13	0.17	0.18	0.12	0.12	0.11	0.16
Val	0.51	0.51	0.55	0.58	0.45	0.46	0.45	0.63
Dispensable AA								
Ala	0.46	0.46	0.44	0.45	0.37	0.38	0.79	0.90
Asp	0.73	0.76	0.63	0.65	0.56	0.57	0.62	1.07
Cys	0.19	0.19	0.29	0.28	0.21	0.19	0.16	0.20
Glu	1.48	1.56	3.69	3.76	1.86	1.88	1.81	2.40
Gly	0.35	0.36	0.51	0.52	0.36	0.37	0.29	0.45
Pro	0.35	0.40	1.27	1.27	0.80	0.87	0.69	0.86
Ser	0.39	0.41	0.55	0.57	0.35	0.36	0.39	0.57
Tyr	0.27	0.26	0.34	0.29	0.22	0.21	0.29	0.39

GE, gross energy; DM, dry matter; OM, organic matter; CP, crude protein; EE, ether extract; NDF, neutral detergent fiber; ADF, acid detergent fiber; TDF, total dietary fiber; SDF, soluble dietary fiber; IDF, insoluble dietary fiber; AA, amino acids.

1) +, Extrusion; −, Non-extrusion.

**Table 2:** Ingredient composition and nutrient levels of the diets in Exp. 1 (%, as-fed basis)^1)^

Item	Broken rice		Wheat		Barley		Sorghum	
			
	−	+	−	+	−	+	−	+
Broken rice	96.90	96.90	-	-	-	-	-	-
Wheat	-	-	96.90	96.90	-	-	-	-
Barley	-	-	-	-	96.90	96.90	-	-
Sorghum	-	-	-	-	-	-	96.90	96.90
Dicalcium phosphate	1.70	1.70	1.70	1.70	1.70	1.70	1.70	1.70
Limestone	0.60	0.60	0.60	0.60	0.60	0.60	0.60	0.60
Salt	0.30	0.30	0.30	0.30	0.30	0.30	0.30	0.30
Premix^2)^	0.50	0.50	0.50	0.50	0.50	0.50	0.50	0.50
Analyzed nutrient levels^3)^								
GE (MJ/kg)	14.95	15.65	16.92	16.98	15.45	15.73	15.71	16.40
DM	87.00	89.64	90.30	92.08	87.72	90.60	87.04	91.01
OM	84.07	87.96	85.18	88.24	83.37	86.35	83.41	85.75
CP	7.68	8.49	14.45	14.64	8.33	9.84	8.96	11.41
EE	0.55	0.26	1.22	1.23	1.17	1.11	1.84	1.00
NDF	2.78	3.02	11.54	10.88	20.86	23.20	9.88	9.99
ADF	-	-	2.99	2.79	4.85	4.53	2.69	2.98
Ash	2.93	2.68	5.12	3.84	4.35	4.25	3.65	5.25

GE, gross energy; DM, dry matter; OM, organic matter; CP, crude protein; EE, ether extract; NDF, neutral detergent fiber; ADF, acid detergent fiber.

1) +, Extrusion; −, Non-extrusion.

2) Premix provided the following per kilogram of feed: vitamin A, 12,000 IU as vitamin A acetate; vitamin D, 2,500 IU as vitamin D_3_; vitamin E, 30 IU as DL-α-tocopheryl acetate; vitamin B_12_, 12 μg; vitamin K, 3 mg as menadione sodium bisulfate; D-pantothenic acid, 15 mg as calcium pantothenate; nicotinic acid, 40 mg; choline, 400 mg as choline chloride; Mn, 30 mg as manganese oxide; Fe, 90 mg as iron sulfate; Cu, 10 mg as copper sulfate; I, 0.35 mg as ethylenediamine dihydroiodide; and Se, 0.3 mg as sodium selenite.

3) The nutrient levels of the diets were analyzed.

**Table 3:** Ingredient composition and nutrient levels of the diets in Exp.2 (%, as-fed basis)^1)^

Item	N-free diet	Broken rice		Wheat		Barley		Sorghum	
			
		−	+	−	+	−	+	−	+
Cornstarch	68.90	-	-	-	-	-	-	-	-
Broken rice	-	96.60	96.60	-	-	-	-	-	-
Wheat	-	-	-	96.60	96.60	-	-	-	-
Barley	-	-	-	-	-	96.60	96.60	-	-
Sorghum	-	-	-	-	-	-	-	96.60	96.60
Sucrose	20.00	-	-	-	-	-	-	-	-
Cellulose acetate	4.00	-	-	-	-	-	-	-	-
Soybean oil	3.00	-	-	-	-	-	-	-	-
Dicalcium phosphate	1.60	1.70	1.70	1.70	1.70	1.70	1.70	1.70	1.70
Limestone	1.00	0.60	0.60	0.60	0.60	0.60	0.60	0.60	0.60
Potassium carbonate	0.30	-	-	-	-	-	-	-	-
Magnesium oxide	0.10	-	-	-	-	-	-	-	-
Salt	0.30	0.30	0.30	0.30	0.30	0.30	0.30	0.30	0.30
Chromic oxide	0.30	0.30	0.30	0.30	0.30	0.30	0.30	0.30	0.30
Premix^2)^	0.50	0.50	0.50	0.50	0.50	0.50	0.50	0.50	0.50
Total	100.00	100.00	100.00	100.00	100.00	100.00	100.00	100.00	100.00
Analyzed nutrient levels^3)^									
CP	0.96	7.98	8.78	12.75	12.18	8.32	8.51	8.53	11.49
Indispensable AA									
Arg	0.01	0.60	0.59	0.57	0.56	0.43	0.42	0.31	0.63
His	0.01	0.18	0.19	0.27	0.28	0.19	0.19	0.19	0.31
Leu	0.02	0.62	0.65	0.77	0.81	0.56	0.58	1.11	1.33
Ile	-	0.32	0.33	0.43	0.44	0.30	0.32	0.36	0.55
Lys	0.02	0.27	0.28	0.34	0.35	0.34	0.34	0.20	0.51
Met	0.01	0.19	0.21	0.18	0.19	0.14	0.14	0.14	0.19
Phe	0.02	0.41	0.46	0.53	0.55	0.41	0.42	0.47	0.64
Thr	0.02	0.28	0.29	0.34	0.36	0.29	0.30	0.28	0.45
Trp	-	0.10	0.12	0.16	0.16	0.11	0.11	0.10	0.17
Val	0.02	0.48	0.49	0.53	0.55	0.45	0.45	0.45	0.66
Dispensable AA									
Ala	0.02	0.43	0.44	0.42	0.43	0.36	0.36	0.76	0.88
Asp	0.03	0.69	0.70	0.60	0.62	0.55	0.55	0.60	1.11
Cys	0.01	0.18	0.19	0.27	0.26	0.21	0.18	0.15	0.22
Glu	0.05	1.43	1.48	3.49	3.67	1.78	1.85	1.78	2.43
Gly	0.01	0.34	0.34	0.49	0.50	0.36	0.36	0.27	0.47
Pro	0.04	0.37	0.36	1.19	1.26	0.85	0.81	0.73	0.86
Ser	0.01	0.38	0.39	0.52	0.55	0.34	0.35	0.38	0.58
Tyr	0.02	0.25	0.25	0.30	0.27	0.23	0.23	0.27	0.36

CP, crude protein; AA, amino acids.

1) +, Extrusion; −, Non-extrusion.

2) Premix provided the following per kilogram of feed: vitamin A, 12,000 IU as vitamin A acetate; vitamin D, 2,500 IU as vitamin D_3_; vitamin E, 30 IU as DL-α-tocopheryl acetate; vitamin B_12_, 12 μg; vitamin K, 3 mg as menadione sodium bisulfate; D-pantothenic acid, 15 mg as calcium pantothenate; nicotinic acid, 40 mg; choline, 400 mg as choline chloride; Mn, 30 mg as manganese oxide; Fe, 90 mg as iron sulfate; Cu, 10 mg as copper sulfate; I, 0.35 mg as ethylenediamine dihydroiodide; and Se, 0.3 mg as sodium selenite.

3) The nutrient levels of the diets were analyzed.

**Table 4:** Effect of extrusion on the available energy and digestibility of cereals (dry matter basis)^1)^

Item		Energy content (MJ/kg)			ATTD (%)						
	
		DE	ME	ME/DE	GE	DM	OM	CP	EE	NDF	ADF
Broken rice	−	17.1	16.9	98.7	95.3	96.6	97.3	85.2	59.3	77.1	-
	+	17.5	17.2	98.7	95.7	96.8	97.6	87.8	30.3	75.9	-
Wheat	−	16.0	15.6	97.8	84.9	89.6	91.3	87.8	35.1	60.3	42.2
	+	16.6	16.1	98.1	87.9	86.4	88.7	85.3	25.1	61.3	41.2
Barley	−	15.0	14.8	98.3	81.1	83.2	83.6	69.6	6.7	57.1	21.5
	+	14.8	14.5	98.3	81.0	83.4	84.0	74.4	4.3	60.1	14.3
Sorghum	−	16.4	16.2	98.8	86.9	89.0	89.8	71.9	30.7	64.7	49.7
	+	16.7	16.5	98.9	88.9	89.5	91.0	80.5	27.8	66.1	56.3
Main effect											
−		16.1	15.9	98.4	87.8	89.6	90.5	78.6	32.9	64.8	37.8
+		16.4	16.2	98.5	87.6	89.0	90.3	82.0	21.9	65.9	37.3
Broken rice		17.2^a^	17.1^a^	98.71	95.5^a^	96.7^a^	97.4^a^	86.5^a^	44.8^a^	76.5^a^	-
Wheat		16.3^b^	16.1^b^	98.0	86.4^b^	88.0^b^	90.0^b^	86.6^b^	30.1^a^	60.8^b^	41.7^a^
Barley		14.9^c^	14.6^c^	98.3	81.0^c^	83.3^c^	83.8^c^	72.0^c^	5.5^b^	58.6^b^	17.9^b^
Sorghum		16.6^b^	16.4^b^	98.9	88.0^b^	89.2^b^	90.4^b^	76.2^b^	29.2^a^	65.4^b^	53.0^a^
SEM		0.13	0.14	0.38	0.74	0.66	0.60	1.62	7.50	4.26	4.77
p-value^2)^											
Ingredient		<0.001	<0.001	0.152	<0.001	<0.001	0.009	<0.001	0.012	<0.001	<0.001
Extrusion		0.352	0.357	0.794	0.770	0.863	0.520	<0.001	0.483	0.443	0.903
Interaction		0.066	0.075	0.619	0.876	0.672	0.786	0.535	0.749	0.945	0.365

DE, digestible energy; ME, metabolizable energy; ME/DE, metabolizable energy/digestible energy; GE, gross energy; DM, dry matter; OM, organic matter; CP, crude protein; EE, ether extract; NDF, neutral detergent fiber; ADF, acid detergent fiber; SEM, standard error of the mean.

1) +, Extrusion; −, Non-extrusion.

2) Comparisons between: Ingredient, broken rice vs wheat vs barley vs sorghum; Extrusion, extrusion vs non-extrusion; Interaction, cereal types and extrusion.

a–c Different superscript in the same column means significantly different (p<0.05).

**Table 5:** Effect of extrusion on the apparent ileal digestibility of crude protein and indispensable amino acids of cereals (%)^1)^

Item		CP	Indispensable AA									

			Arg	His	Leu	Ile	Lys	Met	Phe	Thr	Trp	Val
Broken rice	−	65.6	79.0^abc^	76.8	77.3^ab^	73.3^ab^	68.2^ab^	83.4	77.5	62.0	72.8^ab^	75.2
	+	73.9	87.7^a^	82.3	77.3^ab^	76.9^a^	76.3^a^	77.9	81.4	65.1	71.0^ab^	77.1
Wheat	−	70.1	75.7^bc^	78.3	75.4^ab^	74.0^ab^	57.7^b^	80.4	79.1	55.9	74.2^ab^	69.6
	+	74.4	80.2^abc^	80.9	79.7^ab^	79.2^a^	65.7^ab^	83.8	82.5	66.0	74.4^ab^	74.6
Barley	−	58.1	83.9^ab^	60.4	88.2^a^	51.1^c^	63.2^ab^	65.5	59.1	52.1	56.1^c^	59.1
	+	61.5	81.2^cd^	70.1	87.5^a^	70.3^ab^	62.4^ab^	76.9	74.9	55.2	62.6^bc^	67.3
Sorghum	−	53.7	61.8^d^	58.6	73.8^b^	63.4^bc^	30.5^c^	70.9	70.8	42.6	56.8^c^	59.7
	+	72.0	83.3^ab^	77.4	83.1^a^	79.3^a^	71.7^ab^	81.8	81.5	68.1	77.4^a^	75.7
Main effect												
−		61.9	75.1	68.5	78.7	65.4	54.9	75.0	71.6	53.2	65.0	65.9
+		70.5	80.6	77.7	77.7	76.4	69.0	80.1	80.1	63.6	71.3	73.7
Broken rice		69.8^a^	83.3	79.5^a^	77.3	75.1	72.2	80.7^a^	79.5^a^	63.6	71.9	76.2^a^
Wheat		72.3^a^	77.9	79.6^a^	77.6	76.6	61.7	82.1^a^	80.8^a^	61.0	74.3	72.1^ab^
Barley		59.8^b^	82.5	65.3^b^	87.9	60.7	62.8	71.2^b^	67.0^b^	53.7	59.3	63.2^b^
Sorghum		62.9^ab^	72.5	68.0^b^	78.5	71.3	51.1	76.4^ab^	76.1^ab^	55.4	67.1	67.7^b^
SEM		1.69	1.57	1.86	1.3	1.95	2.62	1.31	1.64	1.92	1.62	1.58
p-value^2)^												
Ingredient		0.004	0.001	<0.001	0.793	0.002	<0.001	0.001	0.002	0.058	<0.001	0.004
Extrusion		<0.001	0.001	<0.001	0.659	<0.001	<0.001	0.003	<0.001	<0.001	<0.001	<0.001
Interaction		0.194	<0.001	0.071	0.008	0.042	<0.001	0.014	0.086	0.065	0.001	0.176

CP, crude protein; AA, amino acids; SEM, standard error of the mean.

Data are least square means of 6 observations per treatment.

1) +, Extrusion; −, Non-extrusion.

2) Comparisons between: Ingredient, broken rice vs wheat vs barley vs sorghum; Extrusion, extrusion vs non-extrusion; Interaction, cereal types and extrusion.

a–d Different superscript in the same column means significantly different (p<0.05).

**Table 6:** Effect of extrusion on the apparent ileal digestibility of dispensable amino acids of cereals (%)^1)^

Item		Dispensable AA						

		Ala	Asp	Cys	Glu	Gly	Ser	Tyr
Broken rice	−	68.2	73.7	76.0^a^	79.3^cd^	49.2	71.1	73.2^ab^
	+	69.9	79.2	70.8^a^	79.0^cd^	69.2	73.4	69.7^ab^
Wheat	−	50.7	60.9	79.9^a^	90.3^ab^	51.2	73.3	75.0^a^
	+	61.5	66.1	78.0^a^	92.3^a^	58.7	77.7	73.7^a^
Barley	−	45.2	58.2	55.1^bc^	80.1^cd^	38.9	61.1	56.1^b^
	+	52.7	59.8	68.2^abc^	83.2^bc^	47.8	62.5	70.8^ab^
Sorghum	−	66.6	58.8	54.9^c^	74.1^d^	48.2	58.7	62.5^b^
	+	76.5	76.2	71.9^ab^	84.3^bc^	56.9	75.5	76.9^a^
Main effect								
−		57.7	62.9	66.5	81.0	46.9	66.1	66.7
+		65.1	70.3	72.2	84.7	58.1	72.3	72.8
Broken rice		69.1^a^	76.4^a^	73.4	79.1	59.2^a^	72.3^ab^	71.4
Wheat		56.1^b^	63.5^b^	78.9	91.3	54.9^ab^	75.5^a^	74.3
Barley		49.0^b^	59.0^b^	61.6	81.7	43.4^b^	61.8^c^	63.4
Sorghum		71.6^a^	67.5^b^	63.4	79.2	52.5^ab^	67.1^ab^	69.7
SEM		2.06	1.78	1.98	1.13	2.35	1.49	1.68
p-value^2)^								
Ingredient		<0.001	<0.001	<0.001	<0.001	0.055	<0.001	0.128
Extrusion		0.001	0.001	0.010	<0.001	0.007	<0.001	0.005
Interaction		0.707	0.291	0.011	0.046	0.503	0.076	0.022

AA, amino acids; SEM, standard error of the mean.

Data are least square means of 6 observations per treatment.

1) +, Extrusion; −, Non-extrusion.

2) Comparisons between: Ingredient, broken rice vs wheat vs barley vs sorghum; Extrusion, extrusion vs non-extrusion; Interaction, cereal types and extrusion.

a–d Different superscript in the same column means significantly different (p<0.05).

**Table 7:** Effect of extrusion on the standardized ileal digestibility of crude protein and indispensable amino acids of cereals (%)^1)^

Item		CP	Indispensable AA									

			Arg	His	Leu	Ile	Lys	Met	Phe	Thr	Trp	Val
Broken rice	−	87.3	86.7^abc^	87.5	88.1^ab^	84.9^ab^	85.8	88.9^ab^	86.4^a^	86.8	88.4	86.4
	+	95.9	96.5^a^	94.0	89.7^ab^	89.9^a^	95.5	85.5^ab^	91.1^a^	91.8	88.3	90.1
Wheat	−	83.7	83.8^bc^	85.4	84.1^b^	82.7^ab^	71.8	86.2^ab^	86.1^a^	75.9	84.0	79.7
	+	88.7	88.5^ab^	87.8	88.1^ab^	87.5^ab^	79.4	89.6^a^	89.1^a^	85.3	84.6	84.4
Barley	−	79.0	92.8^ab^	70.8	93.1^a^	63.3^c^	77.3	73.6^c^	68.0^b^	75.9	70.4	71.1
	+	81.9	92.1^bc^	80.5	92.1^a^	81.9^ab^	76.6	84.7^ab^	83.6^a^	78.5	76.5	79.1
Sorghum	−	74.1	76.5^c^	68.7	79.9^b^	73.7^bc^	54.8	78.3^bc^	78.5^ab^	67.1	72.3	71.7
	+	87.8	91.6^ab^	85.6	89.4^a^	87.6^ab^	83.1	88.3^ab^	88.4^b^	86.3	88.7	85.7
Main effect												
−		81.0	84.9	78.1	88.0	76.1	72.4	81.7	79.7	76.4	78.8	77.2
+		88.6	89.7	87.0	87.3	86.7	83.6	86.9	88.0	85.5	84.5	84.8
Broken rice		91.6^a^	91.6	90.8^a^	88.9	87.4	90.7^a^	87.2	88.8	89.6^a^	88.4^a^	88.2^a^
Wheat		86.2^ab^	86.1	86.6^ab^	86.1	85.1	75.6^b^	87.7	87.6	80.6^ab^	84.3^a^	82.1^ab^
Barley		80.4^b^	92.5	75.7^c^	92.6	72.6	76.9^b^	79.0	75.8	77.2^b^	73.5^b^	75.1^b^
Sorghum		80.9^b^	84.1	77.2^bc^	84.7	80.6	68.9^b^	83.3	83.5	76.7^b^	80.5^ab^	78.7^b^
SEM		1.69	1.57	1.86	1.3	1.95	2.62	1.31	1.64	1.92	1.62	1.58
p-value^2)^												
Ingredient		<0.001	0.014	<0.001	0.173	<0.001	<0.001	<0.001	<0.001	<0.001	<0.001	<0.001
Extrusion		0.010	0.040	0.001	0.640	<0.001	0.001	0.007	<0.001	0.006	0.034	0.003
Interaction		0.948	0.001	0.239	0.007	0.040	0.186	0.008	0.039	0.883	0.185	0.484

CP, crude protein; AA, amino acids; SEM, standard error of the mean.

Data are least square means of 6 observations per treatment.

1) +, Extrusion; −, Non-extrusion.

2) Comparisons between: Ingredient, broken rice vs wheat vs barley vs sorghum; Extrusion, extrusion vs non-extrusion; Interaction, cereal types and extrusion.

a–c Different superscript in the same column means significantly different (p<0.05).

**Table 8:** Effect of extrusion on the standardized ileal digestibility of dispensable amino acids of cereals (%)^1)^

Item		Dispensable AA						

		Ala	Asp	Cys	Glu	Gly	Ser	Tyr
Broken rice	−	84.7	85.6	88.2^a^	85.7	86.8	87.4	83.0
	+	89.1	92.4	84.8^ab^	87.4	71.6	91.7	82.6
Wheat	−	67.8	74.5	88.0^a^	93.0	77.1	85.0	83.1
	+	77.9	79.3	86.2^a^	94.8	83.6	88.9	82.6
Barley	−	62.6	72.9	65.6^c^	85.3	45.5	79.1	66.8
	+	72.3	74.6	80.4^ab^	88.2	71.6	80.0	81.2
Sorghum	−	75.9	72.4	69.3^b^	79.3	64.5	74.9	71.6
	+	86.6	85.5	83.6^ab^	89.6	75.2	88.2	85.1
Main effect								
−		72.8	76.4	74.0	85.8	68.5	81.6	76.1
+		81.5	83.0	83.8	90.0	75.5	87.2	82.8
Broken rice		86.9^a^	89.0^a^	86.5	86.6^b^	79.2	89.6^a^	82.8
Wheat		72.8^bc^	76.9^b^	87.1	93.9^a^	80.4	87.0^ab^	82.8
Barley		67.4^c^	73.8^b^	73.0	86.8^b^	58.5	79.6^b^	74.0
Sorghum		81.3^ab^	79.0^b^	76.4	84.5^b^	69.8	81.5^b^	78.3
SEM		2.06	1.78	1.98	1.13	2.35	1.49	1.84
p-value^2)^								
Ingredient		<0.001	<0.001	<0.001	<0.001	0.025	0.01	0.017
Extrusion		0.011	0.022	0.010	0.005	0.179	<0.010	0.025
Interaction		0.749	0.989	0.001	0.491	0.065	0.93	0.054

AA, amino acids; SEM, standard error of the mean.

Data are least square means of 6 observations per treatment.

1) +, Extrusion; −, Non-extrusion.

2) Comparisons between: Ingredient, broken rice vs wheat vs barley vs sorghum; Extrusion, extrusion vs non-extrusion in main effect; Interaction, cereal types and extrusion.

a–c Different superscript in the same column means significantly different (p<0.05).
